# Otology and Ophthalmology

**Published:** 1880-06

**Authors:** 


### OTOLOGY AND OPHTHALMOLOGY.

COMMUNICATION BETWEEN THE ENDO-AND PERI-LYMPHATIC CAVITIES OF THE LABYRINTH AND EXTRA-LABYRINTHINE INTRACRANIAL CAVITIES.—Weber-Liel: *Virchow's Archiv*, Bd. 77, 1879.—The uncertainty existing in regard to the communications of the two aqueducts of the labyrinth led Weber-Liel to attempt the solution of the question by what he calls the method by aspiration, as well as the old method by injection, and he seems to have met with perfect success in demonstrating that the aquæductus vestibuli is an endo-lymphatic passage in direct and free communication with a sac lying beneath the dura mater on the posterior aspect of the petrous bone, and that the aquæductus cochleæ is a peri-lymphatic passage in free communication with the arachnoid cavity. The sac on the posterior aspect of the petrous bone, saccus intra-duralis or endo-lymphaticus, is of connective tissue, and enclosed in the tissue of the dura mater; it is from 12 to 18 mm. long and from 5 to 9 mm. broad in its normal condition, but is much diminished by pathological changes in the tissues of the dura mater. In sixty preparations it was found in every one, and may be regarded, therefore, as normal. On opening it the interior was lined with flat epithelium, but the question of whether it is in reality a serous sac remains to be settled. From this sac a membranous tube leads directly to the vestibule. To demonstrate that this tube communicated with the endo-lymphatic cavities of the labyrinth, Weber-Liel opened the membranous superior semi-circular canal, attached a glass tube to it, and connected this with an aspirator. The saccus intraduralis was now laid open and filled with "Beale's blue," and suction exerted by the aspirator. The different steps of the operation, with the necessary cautions, are given in full. It was found that by this method both the sacculus of the labyrinth, all the membranous canals, and the ductus cochlearis, were filled with the coloring matter. The very free communication between the saccus intra-duralis and the endo-lymphatic cavities is also shown by the simple experiment of opening the superior semicircular canal so that the endo-lymph is exposed to view, and then pressing or using suction upon the sac, when the endo-lymph will be forced up through the opening in the canal or drawn back out of sight, showing both that the communication is very free, and also that during life any pressure or relaxation in the walls of the sac would influence the tension of the labyrinth itself. On the edges of this sac runs the vena aquæductus vestibuli, and the walls of the sac are surrounded by a network of vessels. To determine the connection of the peri-lymphatic cavities of the labyrinth two methods were used—injection into the arachnoid cavity, and aspiration from that cavity. By the former method the injected fluid entered the scala tympani of the cochlea, and also exuded through the perforated membrane of the fenestra rotunda; but as some of the fluid escaped between the dura and pia mater, it was uncertain whether it had entered the cochlea from the arachnoid cavity, and aspiration was then used, as in the previous experiments. The whole anterior part of the preparation was immersed in the coloring matter, and suction on the opened semicircular canal exerted, with the result of depositing the coloring matter throughout the peri-lymphatic spaces of the labyrinth, but without coloring the porus acousticus internus, thus proving that the connection could not be that canal. The same experiment was tried by first filling the entrance of the aquæductus cochleæ with the blue, and here also the peri-lymphatic spaces only were filled with the coloring matter. The freedom of the communication through the cochlear aqueduct was also shown by filling its orifice with fluid, and then, by condensing and rarefying the air of the meatus, this fluid could be drawn inward or pressed outward. These experiments of Weber-Liel show, then, that the aquæductus vestibuli is a passage communicating and retaining the equilibrium between the endo-lymphatic cavities of the labyrinth and a sac situated within the dura mater, while the aquæductus cochleæ is a passage communicating and retaining the equilibrium between the peri-lymphatic cavities of the labyrinth and the sub-arachnoid cavity. The peculiar sense of pressure in the head (*Eingenommenheit*) experienced with many affections of the ear he would, in some cases, explain by the assumption that the labyrinthine fluids are forced by pressure through these passages into the cranial cavity, and thus exert pressure upon the brain ; and the aural symptoms accompanying some diseases within the cranium he would explain in the opposite way, these passages allowing the fluid to be forced from the arachnoid cavity into the labyrinth. The whole series of experiments and their results are extremely interesting.

EXPERIMENTS ON THE INOCULATION OF THE CORNEA, IRIS, AND CONJUNCTIVA WITH TUBERCLE.—Hænsell, (Græfe's *Archiv*, XXV. 4, p. 1), has studied the changes produced in the cornea, iris, and conjunctiva of rabbits and guinea-pigs by inoculation with the fresh pus from tuberculous joints. This substance, injected into the anterior chamber in an aseptic condition, becomes absorbed in the course of a week. Then, after a period of incubation lasting twenty to thirty days, sudden swelling and hyperæmia of the iris, accompanied by ciliary injection, set in, soon after which small gray points can be seen, which after some time become confluent, press upon, bulge forward, and eventually perforate the cornea, and finally undergo caseous degeneration. The whole process described takes three months in rabbits, in guinea-pigs only about half that time. Inoculation of the cornea directly by interstitial injection of the same pus produces, after fifteen to twenty-three days, severe ulceration with excessive development of blood-vessels, the whole being not unlike a pannus crassus as it occurs in man. Similar results to those which follow injection into the anterior chamber are obtained when the conjunctiva is selected for inoculation. In order to test whether the tubercles formed in the iris and conjunctiva possessed the same power of producing a general tuberculosis as other tubercular substances, they were introduced in two cases into the abdominal cavity, and in these the post-mortem examination, after three months, showed a miliary tuberculosis of all the internal organs. Hænsell has found, with Cohnheim, that any other substance introduced into the anterior chamber is incapable of producing the same effect; on the other hand, it was found possible to infect one animal from the artificially produced iris tubercles of another. In all cases of tubercle of the iris which have been observed in man the same course of events has been noted as in the inoculated tubercle of these animals, only the time taken for complete development is longer. Hænsell considers that the fact of there being a definite period of incubation, as well as that the organisms which Klebs looks upon as being the principal agents in the production of tubercle are constantly found in these neoplasms of the iris, shows that they constitute a truly zymotic disease.

ON THE DISTURBANCE OF VISION CAUSED BY INJURIES TO THE SKULL.—Dr. Berlin has collected a number of cases which have come under the observation of himself and others, in which amaurosis has followed severe blows on the head. In an interesting paper, read before the Ophthalmological Society at Heidelberg, he has given his views on this subject. These may briefly be summed up as follows: The amaurosis is in by far the greatest number of cases unilateral; it arises suddenly at the time of, or immediately after the injury, and is generally complete and incurable. The injuries giving rise to the amaurosis are almost always severe, such, indeed, as generally give rise to loss of consciousness at the time, and may often be supposed to have caused fracture. The frequency of fracture of the roof of the orbit is very great, as may be seen from the statistics of Hölder. Of 126 cases of fracture of the base of the skull examined by him, 88 involved the base, in eighty, or ninety per cent., of which the roof of the skull was fractured. In fifty-four, or sixty per cent., the line of fracture passed through the walls of the foramen opticum, and this independent of the direction of the force producing the fracture. In the 54 fractures of the optic foramen Hölder found extravasation of blood into the sheath of the optic nerve 42 times. No extravasation into the sheath of the nerve was found in any case when the fracture did not involve the foramen. Taking into consideration, then, the severe nature of the injuries causing amaurosis, and the statistics of Hölder, Berlin is of opinion that the amaurosis in such cases is generally the result of injury to the optic nerve from fracture through the foramen.

REMOVAL OF A FOREIGN BODY FROM THE VITREOUS CHAMBER BY MEANS OF A MAGNET.—Hirschberg removed successfully a piece of iron 3 mm. long and 2 mm. broad from the vitreous chamber with an electro-magnet which he had constructed for the purpose. An incision was made in the sclerotic near where the foreign body was seen with the ophthalmoscope to lie. The magnet used can attract pieces of iron of 1 to 5 mm. in length from a distance of 2 to 4 mm. when placed free in vitreous humor. He concludes his account of the case with the following remarks on this method of operating: "The asserted possibility of removing with a magnet a piece of iron embedded in the cornea, as we see them daily in iron-workers, and which are so easily removed by mechanical means, must be looked upon as a fable, a counterpart to the tale in the Arabian Nights, of the magnetic island which attracted the nails from the planks of passing ships. For foreign bodies in the anterior chamber, the magnet is as a rule, superfluous and impractical; as soon as the aqueous humor escapes, and the piece of iron is pressed up against the posterior surface of the cornea, it can easily be extracted with forceps, whilst extraction with a magnet is exceedingly difficult, as will be found by experimenting on rabbits. The triumphant cases for the magnet are only those in which, as in our case, the piece of iron has recently found its way into the eye and lies free in the vitreous; in such cases it could only rarely be extracted with forceps or scoop without producing lasting injury to the eye."—*Centralblatt f. Augenh.*, December, 1879.

ON THE SENSIBILITY OF THE ORGAN OF HEARING.—W. Kohlrausch. (Wiedemann's *Annalen*, 1879, No. 6; *Phil. Mag.*, No. 48, September.) With the aid of a toothed wheel working agaist a disk of metal or pastboard, Savart found that two impulses can make upon the ear the impression of a comparable tone. Exner (Pfluger's *Archiv*, XIII. p. 228), by means of tuning-forks vibrating before spherical resonators, finds that seventeen impulses—Pfaundler (*Wiener Berichte*, LXXVI. p. 572, 1877), by experiments on holed sirens and reflection-tones, that only two impulses—and Auerbach (Wiedemann's *Annalen*, VI. p. 591, 1879), that about twenty vibrations are necessary for the production of a tone in the physiological sense, the tone being determined to within the interval 100 : 101. Less sharply defined tones can be produced in a simple manner by but two impulses. Two fingers of the hand being held together so that the ends of the finger-nails are on a level, a blow is given therewith somewhat obliquely upon a table or board, the proper tone of which is deadened by loading it with books or some other heavy substances. It will be readily felt that the two fingers rarely strike simultaneously, and by a little attention there will be heard, with the noise of the blow, a very hollow tone of a pitch, which changes *per saltum* according to the position of the fingers, but which, by practice, can be approximately had at command. Similar tones are obtained by running the finger-nails over short lengths of ribbed paper.

A RARE FORM OF OPACITY OF THE CORNEA.—In this paper (Knapp's *Archiv*, IX. 2, p. 184) Dr. Nettleship gives an excellent description of a traverse opacity, which occurs rarely and under circumstances still imperfectly understood, in the cornea of old people. The description given differs slightly from that given by Græfe in one of his well-known papers on glaucoma. Whereas Græfe seems always to have observed the opacity to begin in two separate portions at the inner and outer margins of that part of the cornea which is uncovered by the lids, and gradually ex-extend until they met over the center of the pupil, Nettleship has seen the process begin as frequently in the center, and is of opinion that the margins of the cornea always remain free. Further, Græfe has associated these cases constantly, sooner or later, with secondary glaucoma, whilst Nettleship believes that they are sometimes primary, uncomplicated corneal changes. A number of valuable suggestions are thrown out as to the possible etiology of this curious condition. These are preceded by a careful examination of all the causes which might be supposed to influence its occurrence in any way. The opinion of the author seems to be that some constitutional or trophic conditions favor the gradual alteration in the most exposed part of the cornea, where the opacity is invariably situated. The treatment advocated and adopted with success is the scraping away of the opacity from the center of the pupil. A recurrence after this operation does not appear to take place. It will be remembered that V. Græfe recommended iridectomy.

ON THE CHORDA TYMPANI NERVE.—Horatio B. Bigelow. (*Archives of Medicine*, June, 1879.) The author presents the result of an anatomical and physiological investigation of the chorda tympani nerve in rabbits and dogs, instituted during the spring and summer of 1875. He is led to take issue with the views entertained by Prof. Sappey (*Traité d'Anatomie descriptive*) respecting the course and function of this nerve. He says: "To me there seems to be no doubt that the special sensory function of the facial filaments is derived from the nerve-cells in the intumescentia gangliformis found upon the nerve of Wrisberg in the aquæductus Fallopii. The chorda tympani sends branches to the lingual just after becoming joined to it in the common sheath between the pterygoid muscles. It is so well nigh impossible to separate these connecting filaments, and so closely apposed to each other are the two trunks, that I am perfectly aware of the unsatisfactory nature of the dissections; but yet, by means of the glass, I was able to identify so many branches of the chorda tympani passing to the lingual, and so satisfactorily, for a considerable distance, demonstrated the integrity of each nerve, that I feel convinced that at no very distant day it will be proven conclusively that these nerves are not joined fibril to fibril, but that they pass together in one sheath, the lingual receiving branches from the chorda tympani, which, in turn, is made sensory through *the ganglion upon the nerve of Wrisberg.*"—*American Journal of Otology*, Vol. II, No. 1.

